# Early and Late Postnatal Accelerated Growth Have Distinct Effects on Metabolic Health in Normal Birth Weight Infants

**DOI:** 10.3389/fendo.2017.00340

**Published:** 2017-12-04

**Authors:** Dan-Li Zhang, Qinwen Du, Anissa Djemli, Pierre Julien, William D. Fraser, Zhong-Cheng Luo

**Affiliations:** ^1^Ministry of Education-Shanghai Key Laboratory of Children’s Environmental Health, Xinhua Hospital, Shanghai Jiao-Tong University School of Medicine, Shanghai, China; ^2^Department of Obstetrics and Gynecology, Sainte-Justine Hospital Research Center, University of Montreal, Montreal, Canada; ^3^Department of Clinical Biochemistry, Sainte-Justine Hospital Research Center, University of Montreal, Montreal, Canada; ^4^Department of Pediatrics, Sainte-Justine Hospital Research Center, University of Montreal, Montreal, Canada; ^5^Department of Medicine, CHU de Québec-Université Laval Research Center, Quebec City, Canada; ^6^Department of Endocrinology, CHU de Québec-Université Laval Research Center, Quebec City, Canada; ^7^Department of Nephrology, CHU de Québec-Université Laval Research Center, Quebec City, Canada; ^8^Department of Obstetrics and Gynecology, University of Sherbrooke, Sherbrooke, Canada; ^9^Lunenfeld-Tanenbaum Research Institute, Mount Sinai Hospital, University of Toronto, Toronto, ON, Canada

**Keywords:** infant, postnatal accelerated growth, insulin sensitivity, beta-cell function, fasting blood cholesterols

## Abstract

Accelerated growth in postnatal life in low birth weight infants has been associated with insulin resistance and metabolic syndrome-related disorders in later life. Postnatal accelerated growth in also common in normal birth weight infants, but little is known about the impact on metabolic health. In a prospective cohort study of 203 term normal birth weight infants, we evaluated the impacts of accelerated (Δweight *Z* score > 0.5) or decelerated (Δweight Δ*Z* < −0.5) growth during early (0–3 months) and late (3–12 months) postnatal life on metabolic health indicators at age 1-year. The primary outcomes were homeostasis model assessment of insulin resistance (HOMA-IR), β-cell function [homeostasis model assessment of β-cell function (HOMA-β)], and fasting plasma lipids. Adjusting for maternal, paternal, and infant characteristics, accelerated growth during the first 3 months of life was associated with a 41.6% (95% confidence interval 8.9–84.2%) increase in HOMA-β, and a 8.3% (0.7–15.4%) decrease in fasting plasma total cholesterols, and was not associated with HOMA-IR in infants at age 1-year. Accelerated growth during 3–12 months was associated with a 30.9% (3.3–66.0%) increase in HOMA-IR and was not associated with HOMA-β. Neither accelerated nor decelerated growth was associated with fasting plasma triglycerides, high-density lipoprotein or low-density lipoprotein cholesterol concentrations in infants at age 1-year. Accelerated growth during early postnatal life may be beneficial for β-cell function, but during late postnatal life harmful for insulin sensitivity in normal birth weight infants.

## Introduction

Preterm and/or low birth weight infants often have postnatal catch-up or accelerated growth (usually defined as Δweight *Z* score > 0.5 or 0.67) which has been consistently associated with insulin resistance and metabolic syndrome-related disorders (e.g., type 2 diabetes) in later life (1–8). Accelerated growth during early postnatal life (the first 2–4 months) appears to be particularly important for metabolic health in later life (5, 9, 10), but whether the adverse metabolic health impact may be evident as early as in infancy is unclear.

Although low birth weight subjects are at elevated risk (about 1.5-fold) of type 2 diabetes in adulthood (11), the vast majority of patients with type 2 diabetes are not low birth weight. It is now increasingly recognized that adverse metabolic programming in early life may occur irrespective of birth weight (12). Considering the adverse metabolic health impact of postnatal accelerated growth in low birth weight subjects, it is plausible that such accelerated growth may also have an adverse metabolic health impact in normal birth weight infants. Accelerated or decelerated postnatal growth is common in normal birth weight infants, yet there is a scarcity of data on the metabolic health impact in these infants. It is unknown whether the metabolic health impact may be different for accelerated growth during early vs. late postnatal life in the first year of life, a rapid growth period which may be critical for long-term metabolic health. Studies have been focused on the metabolic health impact of postnatal accelerated growth, and there is a lack of data on postnatal decelerated growth. To address these data gaps, we sought to determine whether accelerated or decelerated growth during early (0–3 months) or late (3–12 months) postnatal period is associated with metabolic health indicators in infants at age 1-year.

## Materials and Methods

### Study Population

**Figure 1:**
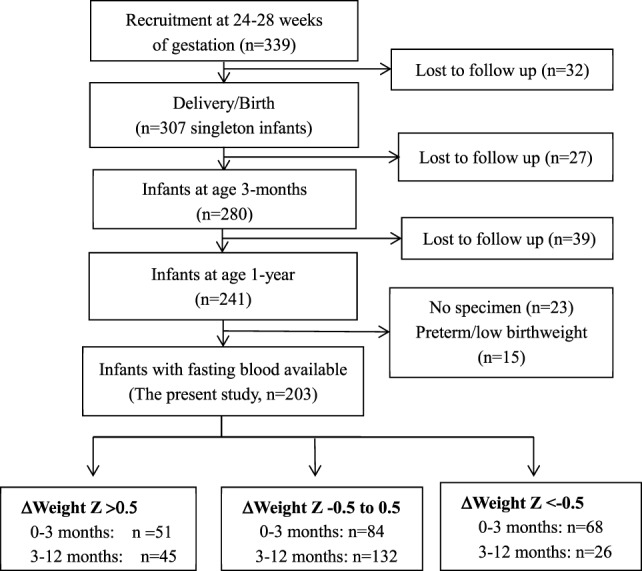
Selection of study participants in a birth cohort.

This study was based on a prospective pregnancy cohort study described previously (13–16). Here, we presented the new data on infant follow-ups to assess the impacts of postnatal accelerated or decelerated growth on metabolic health indicators in infants. Briefly, 339 healthy women bearing a singleton non-malformation fetus without pre-existing diabetes, chronic hypertension, endocrine disorders, or other severe maternal illnesses were recruited at 24–28 weeks of gestation between August 2006 and December 2008 in three obstetric care centers in Montreal. The women were followed up at delivery (*n* = 307), and the infants were followed up at 3 months (*n* = 280) and 1 year (*n* = 241) of age. This study included all 203 normal birth weight babies with postnatal growth data and fasting infant blood specimen available for biomarker assays (Figure 1). The study was approved by the research ethics committee of Sainte-Justine hospital research center, University of Montreal. Written informed consent was obtained from all study participants.

### Data and Specimen Collections in Infant Follow-ups

Data and specimen collections up to delivery were described previously (13). Here, we described the infant follow-up data and specimen collection procedures.

At 3 and 12 months (1-year) of age, infants were followed up for data collection on feeding and growth measurements. Infant feeding was classified as any breastfeeding or non-breastfeeding. Infant’s length was measured in supine position by the 447 Infantronic Digital Infantometer (QuickMedical, Seattle, WA, USA) to the nearest 0.1 cm. Weight was measured by an electronic weighting scale to the nearest gram (g). Skinfold thickness at triceps and subscapular positions was measured by a Harpenden skinfold caliper (Baty International, West Sussex, England) to the nearest 0.1 mm. All anthropometric measurements were taken twice, and the average values were used in the final analysis data. All follow-ups and anthropometric measurements were done by a trained pediatric research nurse.

#### Accelerated or Decelerated Growth

Weight and length *z* scores (for sex and gestational age) at birth, 3, and 12 months of age were calculated using the WHO child growth standards (17). We calculated the changes (Δ) in weight *z* scores between 0–3 and 3–12 months of age. Accelerated growth was defined as a 0.5 or greater increase in weight *z* scores between 0–3 and 3–12 months of age (5). Similarly, decelerated growth was defined as a 0.5 or greater decrease in weight *z* scores between 0–3 and 3–12 months of age.

#### Infant Blood Sampling

At 12 months of age, a morning fasting blood sample was collected from the infant. A 0.5-ml fluoride (stopping glucose oxidation) tube of blood was specifically collected for the glucose assay. The blood specimens were kept on ice, and centrifuged within 30 min after specimen collection. The separated plasma samples were stored in multiple aliquots in a −80°C freezer until assays.

### Biochemical Assays

The assays of plasma glucose and insulin followed the protocols described previously (13). Plasma lipids [triglycerides, high-density lipoprotein (HDL), low-density lipoprotein (LDL), and total cholesterols (TC)] (mmol/l) were measured with an automated multianaylser (Unicel DXC 880i, Beckman Coulter). The intra-assay and inter-assay coefficient variations of these assays were lower than 3.0%. All biochemical assays were completed in the Sainte-Justine clinical biochemistry lab at 12–18 months after the specimen collection. There were no significant correlations between storage time and measurement values in all measured biomarkers (all *p* > 0.1).

### Outcomes

The primary outcomes were homeostasis model assessment of insulin resistance (HOMA-IR) and β-cell function [homeostasis model assessment of β-cell function (HOMA-β)] and fasting plasma lipids (TC, triglycerides, HDL, and LDL cholesterols) in infants at age 1-year. HOMA-IR was calculated as fasting insulin (mU/l)*fasting glucose (mmol/l)/22.5, HOMA-β as fasting insulin (mU/l)/[fasting glucose (mmol/l) −3.5] (18). Other outcomes included weight-for-length *z* score [WHO child growth standards (17)], body mass index (BMI) and skinfold thickness (triceps and subscapular positions) as indicators of body fat (19).

### Statistical Analysis

The primary exposures of interest were accelerated or decelerated growth during 0–3 and 3–12 months of age. The co-variables included maternal height (SD score), pre-pregnancy BMI (SD score), biological father’s height (SD score) and BMI (SD score), maternal family history of diabetes (yes/no, among first-degree relatives), ethnicity (White, other), age (maternal age <35 and ≥35 years), parity (primiparous: yes/no), prenatal smoking (yes/no), alcohol use (yes/no), gestational diabetes (yes/no), gestational hypertensive disorders (yes/no), mode of delivery (cesarean, vaginal), infant sex, gestational age (weeks), birth weight (*z* score), and breast feeding (yes/no). Biomarker variables (positively skewed crude data distributions) were log-transformed in the comparisons for differences between groups. Generalized linear regression models were used to assess the differences adjusting for multiple co-variables. For log-transformed biomarker outcomes, we calculated the adjusted % differences in the original scale between two groups according to the regression coefficients. The sample size is sufficient in multivariate regression models since linear models require a minimal of two subjects per variable (20). Data management and analyses were conducted using Statistical Analysis System (SAS), Version 9.4 (SAS Institute, Cary, NC, USA). Two-tailed *p* values <0.05 were considered statistically significant.

## Results

### Parental, Pregnancy, and Infant Characteristics

Table 1 shows maternal, pregnancy, and infant characteristics of the birth cohort (*n* = 203). The majority of mothers were white (about 70%). About a quarter of mothers were older than 35 years, and 17% of mothers had a family history of diabetes. The mean pre-pregnancy BMI was 23.8 kg/m^2^. The mean birth weight was 3,458 g. About 90% of infants were breast-fed. The average changes in weight *z* scores were close to 0 during both 0–3 and 3–12 months of postnatal age.

**Table 1:** Parental, pregnancy, and infant characteristics of the study birth cohort (*n* = 203).

Characteristic	Median, mean ± SD or *n* (%)
**Mothers**	
Ethnicity: white	142 (70.0)
Age (years)	31.0, 31.1 ± 4.6
Family history of diabetes	34 (16.8)
Gestational diabetes	11 (5.4)
Gestational hypertension	10 (4.9)
Height (cm)	165.0, 164.6 ± 6.3
Pre-pregnancy BMI	22.4, 23.8 ± 4.9
**Fathers**	
Height (cm)	177.0, 177.7 ± 7.4
BMI (kg/m^2^)	25.6, 26.3 ± 4.0
**Newborns**	
Gender, male	106 (52.2)
Gestational age (weeks)	39.0, 39.2 ± 1.4
Birth weight (*z* score)	0.04, 0.08 ± 0.9
Birth weight (g)	3,450, 3,458 ± 403
Birth length (cm)	51.0, 50.7 ± 2.1
**Infant growth**	
ΔWeight *Z*, 0–3 months	−0.11, −0.07 ± 0.9
3–12 months	0.12, 0.15 ± 0.7
**Infants at 3 months**	
Breastfeeding	184 (90.1)
Weight (kg)	6.4, 6.3 ± 0.7
Length (cm)	61.7, 61.7 ± 2.3
(T + S) skinfold (mm)	16.6, 16.7 ± 3.2
**Infants at 12 months**	
Weight (kg)	9.8, 9.9 ± 1.5
Length (cm)	76.4, 76.6 ± 3.1
(T + S) skinfold (mm)	15.9, 16.1 ± 3.1
BMI (kg/m^2^)	17.0, 16.9 ± 1.6
**Fasting blood (1-year)**	
Glucose, mmol/l	4.5, 4.5 ± 0.6
Insulin, pmol/l	20.8, 25.6 ± 16.8
HOMA-IR	0.7, 0.9 ± 0.7
HOMA-β, %	74.0, 96.3 ± 77.9
Lipids (mmol/l)	
Triglycerides	1.0, 1.1 ± 0.5
HDL	1.2, 1.2 ± 0.3
LDL	2.7, 2.8 ± 0.8
TC	4.4, 4.4 ± 0.9

*Data presented are median, mean ± SD for continuous variables, and *n* (%) for frequency variables*.

*(T + S) skinfold, sum of triceps and subscapular skinfold thickness (mm); BMI, body mass index (kg/m^2^); HOMA-IR, homeostasis model assessment of insulin resistance; HOMA-β, homeostasis model assessment of β-cell function; TC, total cholesterols; LDL, low-density lipoprotein; HDL, high-density lipoprotein*.

### Infant Metabolic Health Biomarkers by Postnatal Growth Pattern

Table 2 presents the descriptive statistics on BMI, skinfold thickness, HOMA-IR, HOMA β, and fasting blood TC, triglycerides, LDL, HDL cholesterol concentrations in infants at age 1-year stratified by postnatal growth pattern (accelerated, normal, or decelerated). Compared to infants with normal weight gains, infants with accelerated growth during 0–3 or 3–12 months of life age had higher skinfold thickness at age 1-year, while those with decelerated growth during 3–12 months of age had lower skinfold thickness. Infants who had accelerated growth during 3–12 months of age had higher HOMA-IR at age 1-year. There were no differences in fasting plasma lipids between infants with accelerated or decelerated growth vs. infants with normal weight gain.

**Table 2:** Metabolic health outcomes in infants at age 1-year by postnatal growth pattern (accelerated, normal, and decelerated) during 0–3 and 3–12 months of age (*n* = 203).

	ΔWeight *Z* 0–3 months			ΔWeight *Z* 3–12 months		
Outcome	Accelerated	Normal	Decelerated	Accelerated	Normal	Decelerated
		
	>0.5	−0.5 to 0.5	<−0.5	>0.5	−0.5 to 0.5	<−0.5
BMI, kg/m^2^	17.4 ± 0.2	16.9 ± 0.2	**16.5 ± 0.2**^c^	**17.6 ± 0.2**^a^	16.7 ± 0.1	16.3 ± 0.3
Weight-for-length, *z* score	**0.8 ± 0.1**^a^	0.3 ± 0.1	**−0.04 ± 0.1**^b^	**0.9 ± 0.2**^a^	0.2 ± 0.1	−0.1 ± 0.2
(T + S) skinfold, mm	**17.5 ± 0.4**^a^	15.6 ± 0.3	15.7 ± 0.4	**17.2 ± 0.5**^b^	16.0 ± 0.3	**15.0 ± 0.5**^c^
HOMA-IR	0.92 ± 0.08	0.88 ± 0.07	0.85 ± 0.08	**1.1 ± 0.1**^c^	0.82 ± 0.05	0.85 ± 0.1
HOMA-β, %	118.3 ± 15.6	86.4 ± 7.1	92.1 ± 7.6	101.1 ± 14.4	96.2 ± 6.8	90.4 ± 11.1
Lipids, mmol/l						
TC	4.2 ± 0.1	4.5 ± 0.1	4.4 ± 0.08	4.5 ± 0.1	4.4 ± 0.08	4.3 ± 0.2
Triglycerides	1.0 ± 0.07	1.1 ± 0.06	1.05 ± 0.05	1.1 ± 0.1	1.0 ± 0.04	1.2 ± 0.2
LDL	2.6 ± 0.1	2.9 ± 0.1	2.8 ± 0.07	2.8 ± 0.1	2.8 ± 0.07	2.7 ± 0.2
HDL	1.1 ± 0.04	1.2 ± 0.03	1.2 ± 0.03	1.2 ± 0.04	1.2 ± 0.02	1.1 ± 0.1

*Data presented are mean ± SE*.

*^a^*p* < 0.001, ^b^*p* < 0.01, ^c^*p* < 0.05, compared to the normal (Δ*Z* score −0.5 to 0.5) reference group, in the analysis of variance tests for differences; the log-transformed data were used for biomarkers in the comparisons; data in bold font: *p* < 0.05 compared to the normal growth reference group*.

*(T + S) skinfold, sum of triceps and subscapular skinfold thickness (mm); HOMA-IR, homeostasis model assessment of insulin resistance; HOMA-β, homeostasis model assessment of β-cell function; TC, total cholesterol; LDL, low-density lipoprotein; HDL, high-density lipoprotein; BMI, body mass index*.

Table 3 presents the changes (95% CI) in metabolic health outcomes at age 1-year comparing infants with accelerated or decelerated growth to infants with normal growth adjusted for maternal, paternal and infant characteristics. Accelerated growth during the first 3 months of life was associated with a 41.6% (95% CI: 8.9–84.2%) increase in HOMA-β (*p* = 0.01), an 8.3% (0.7–15.4%) decrease in fasting plasma TC (*p* = 0.03), and a 2.7 (1.6, 3.7) mm increase in the sum of triceps and subscapular skinfold thickness, but was not associated with HOMA-IR in infants at age 1-year. Accelerated growth during 3–12 months of life was associated with a 30.9% (3.3–66.0%) increase in HOMA-IR (*p* = 0.03), and a 1.5 (0.5, 2.5) mm increase in the sum of triceps and subscapular skinfold thickness, but was not associated with HOMA-β. As expected, during both 0–3 and 3–12 months of age, accelerated growth was associated with higher BMI and weight-for-length *z* scores, while decelerated growth was associated with lower BMI and weight-for-length *z* scores in infants at age 1-year.

**Table 3:** Adjusted changes (%)^d^ in infant metabolic health outcomes in infants at age 1-year comparing accelerated or decelerated growth to normal growth infants during 0–3 and 3–12 months of age (*n* = 203).

	ΔWeight *Z* 0–3 months		ΔWeight *Z* 3–12 months	
Outcome^d^	Accelerated	Decelerated	Accelerated	Decelerated
		
	>0.5	<−0.5	>0.5	<−0.5
BMI, kg/m^2^	**1.1 (0.6, 1.5)**^a^	**−1.0 (−1.5, −0.6)**^a^	**1.0 (0.5, 1.5)**^a^	**−1.0 (−1.6, −0.4)**^a^
Weight-for-length, *z* score	**0.9 (0.6, 1.2)**^a^	**−0.8 (−1.1, −0.6)**^a^	**0.8 (0.6, 1.1)**^a^	**−0.8 (−1.1, −0.4)**^a^
(T + S) skinfold, mm	**2.7 (1.6, 3.7)**^a^	−0.7 (−1.7, 0.3)	**1.5 (0.5, 2.5)**^c^	**−1.5 (−2.8, −0.2)**^c^
HOMA-IR	12.7 (12.8, 45.5)	−0.5 (−22.0, 27.1)	**30.9 (3.3, 66.0)**^c^	−12.9 (−36.4, 19.3)
HOMA-β	**41.6 (8.9, 84.2)**^c^	−5.2 (−26.4, 22.0)	−1.6 (−22.9, 25.7)	−11.1 (−35.9, 23.1)
TC	**−8.3 (−15.4, −0.7)**^c^	−1.3 (−8.7, 6.6)	4.9 (−2.8, 13.3)	2.6 (−7.0,13.1)
Triglycerides	−8.3 (−20.8, 6.2)	−2.8 (−15.7, 12.0)	12.8 (−2.0, 29.8)	18.7 (−0.8, 42.1)
LDL	−9.5 (−19.2, 1.4)	−1.8 (−12.0, 9.6)	4.5 (−6.2, 16.6)	0.3 (−12.7, 15.3)
HDL	−4.7 (−13.0, 4.5)	0.3 (−8.2, 9.7)	4.7 (−4.2, 14.3)	−4.7 (−14.8, 6.6)

*^a^*p* < 0.001, ^b^*p* < 0.01, ^c^*p* < 0.05, compared to the “normal” (Δ*Z* score −0.5 to 0.5) reference group; data in bold font: *p* < 0.05 compared to the normal growth reference group*.

*^d^Data presented are the adjusted % changes for biomarkers (HOMA-IR, HOMA-β, lipids), in mm for skinfold thickness, in kg/m^2^ for BMI, as compared to the reference normal growth group (Δ*Z* score −0.5 to 0.5). The effect estimates were adjusted for parental and infant characteristics including maternal ethnicity, age, parity, height, pre-pregnancy BMI, prenatal smoking, alcohol use, family history of diabetes, gestational diabetes, gestational hypertension, biological father’s height and BMI, infant sex, mode of delivery, gestational age (weeks), birth weight (*z* score), and breast feeding, based on generalized linear models*.

*(T + S) skinfold, sum of triceps and subscapular skinfold thickness (mm); HOMA-IR, homeostasis model assessment of insulin resistance; HOMA-β, homeostasis model assessment of β-cell function; TC, total cholesterol; LDL, low-density lipoprotein; HDL, high-density lipoprotein; BMI, body mass index*.

## Discussion

### Main Findings

Our study is the first to reveal potentially differential metabolic health impacts of accelerated growth during early vs. late postnatal periods during the first year of life in normal birth weight infants. Accelerated growth during the first 3 months appears to be beneficial for β-cell function, but during 3–12 months harmful for insulin sensitivity in infants at age 1-year.

### Postneonatal Accelerated Growth and Metabolic Health

There is a scarcity of data on the relationship between accelerated growth, insulin sensitivity, and β-cell function in infancy. We are aware of only one study—Soto and colleagues studied 85 small for gestational age (SGA) and 23 birth weight appropriate for gestational age (AGA) infants, and found that accelerated growth in SGA infants during the first year of life was associated with increased insulin resistance (21). In contrast, in a prospective cohort of modest sample size (*n* = 203), we first discovered the timing-dependent impacts of accelerated growth in early (first 3 months) vs. late postnatal (3–12 months of age) periods on metabolic health in infancy. Accelerated growth during the first 3 months of life was associated with higher β-cell function and lower fasting blood TC, but during 3–12 months of life was not. Crude mean HOMA-β was 31.9% higher in infants with accelerated growth compared to those with normal growth during the first 3 months of life (mean: 118.3 vs. 86.4%). This difference did not reach statistically significance (*p* = 0.11) in the crude comparison (Table 2), but became significant (*p* = 0.01) after the adjustments (Table 3), suggesting that the crude comparison was clouded by confounding factors. In contrast, accelerated postnatal growth during 3–12 months of life was associated with higher insulin resistance.

As expected, accelerated growth during either early or late postnatal period was associated with greater skinfold thickness at age 1-year. Accelerated weight gain may come along with some fat deposition in subcutaneous tissue.

### Postneonatal Decelerated Growth and Metabolic Health

We are unaware of any research data on the relationship between decelerated growth and metabolic health in normal birth weight infants. As expected, decelerated growth in either the 0–3 or 3–12 months was associated with lower BMI at age 1-year. Decelerated growth during 3–12 months was also associated with lower skinfold thickness. However, decelerated growth in either period was not associated with HOMA-IR or HOMA-β.

### Are the Findings Consistent with Evidence in Animal Models?

The observed positive association between early postnatal accelerated growth and HOMA-β in infant at age 1-year is consistent with the findings in animal studies. It has been shown that insulin secretion in 20-day-old mice was decreased significantly as a consequence of restricted nutrient intake during the suckling period (22), and greater weight gain during the suckling period could improve β-cell function in neonatal mice (23).

### Strengths and Limitations

Strengths of the study include the high rates of infant follow-up and fasting blood specimen collection, and the analyses accounting for prenatal and postnatal potential confounding factors. One limitation is that the HOMA insulin resistance and β-cell function indices are based on glucose and insulin concentrations in a single fasting blood sample. Studies using more invasive techniques with multiple blood samplings (e.g., intravenous glucose tolerance test) may yield more accurate estimates of insulin resistance and β-cell function, but such procedures are not so acceptable to most parents of small infants. However, HOMA-IR and HOMA-β have been validated against the gold standard methods—the euglycemic–hyperinsulinemic clamp and first-phase insulin secretion on intravenous glucose tolerance test in children (24). The study was based on a Canadian birth cohort (the majority are Caucasians). Further studies in other countries/regions are required to understand the generalizability of the study findings.

## Conclusion

Accelerated growth during early postnatal life (the first 3 months) may be beneficial for β-cell function, but during late postnatal life (3–12 months) detrimental for insulin sensitivity in normal birth weight infants at age 1-year. The findings suggest differential impacts of early vs. late postnatal accelerated growth on metabolic health in normal birth weight infants.

## Ethics Statement

This study complies with the guidelines of the Declaration of Helsinki. Written informed consent has been obtained from each study participant. The study was approved by the research ethics board of Sainte-Justine Hospital Research Center, University of Montreal.

## Author Contributions

Z-CL, PJ, and WF developed the research protocol and obtained the research grants; D-LZ, QD, AD, PJ, WF, and Z-CL contributed to the acquisition of research data; D-LZ, QD, and Z-CL contributed to the data analyses; D-LZ drafted the manuscript. All authors contributed to improvements of the manuscript for important intellectual content and approved the final version for publication.

## Conflict of Interest Statement

The authors declare that the research was conducted in the absence of any commercial or financial relationships that could be construed as a potential conflict of interest.

## Abbreviations

HOMA-IR, homeostasis model assessment of insulin resistance; HOMA-β, homeostasis model assessment of β-cell function; TC, total cholesterols; LDL, low-density lipoprotein; HDL, high-density lipoprotein; (T + S) skinfold, the sum of triceps and subscapular skinfold thickness.

## References

[1] Ong KK Ahmed ML Emmett PM Preece MA Dunger DB. Association between postnatal catch-up growth and obesity in childhood: prospective cohort study. BMJ (2000) 320:967–71. 10.1136/bmj.320.7240.967 10753147 PMC27335

[2] Singhal A Cole TJ Fewtrell M Kennedy K Stephenson T Elias-Jones A Promotion of faster weight gain in infants born small for gestational age: is there an adverse effect on later blood pressure? Circulation (2007) 115:213–20. 10.1161/circulationaha.106.617811 17179023

[3] Ekelund U Ong KK Linne Y Neovius M Brage S Dunger DB Association of weight gain in infancy and early childhood with metabolic risk in young adults. J Clin Endocrinol Metab (2007) 92:98–103. 10.1210/jc.2006-1071 17032722

[4] Chomtho S Wells JC Williams JE Davies PS Lucas A Fewtrell MS. Infant growth and later body composition: evidence from the 4-component model. Am J Clin Nutr (2008) 87:1776–84. 18541568 10.1093/ajcn/87.6.1776

[5] Leunissen RW Kerkhof GF Stijnen T Hokken-Koelega A. Timing and tempo of first-year rapid growth in relation to cardiovascular and metabolic risk profile in early adulthood. JAMA (2009) 301:2234–42. 10.1001/jama.2009.761 19491185

[6] Singhal A Cole TJ Fewtrell M Deanfield J Lucas A. Is slower early growth beneficial for long-term cardiovascular health? Circulation (2004) 109:1108–13. 10.1161/01.cir.0000118500.23649.df 14993136

[7] Bavdekar A Yajnik CS Fall CH Bapat S Pandit AN Deshpande V Insulin resistance syndrome in 8-year-old Indian children: small at birth, big at 8 years, or both? Diabetes (1999) 48:2422–9. 10.2337/diabetes.48.12.2422 10580432

[8] Eriksson JG Early growth and coronary heart disease and type 2 diabetes: findings from the Helsinki Birth Cohort Study (HBCS). Am J Clin Nutr (2011) 94:1799s–802s. 10.3945/ajcn.110.000638 21613556

[9] Singhal A Cole TJ Lucas A. Early nutrition in preterm infants and later blood pressure: two cohorts after randomised trials. Lancet (2001) 357:413–9. 10.1016/s0140-6736(00)04004-6 11273059

[10] Kerkhof GF Willemsen RH Leunissen RW Breukhoven PE Hokken-Koelega AC. Health profile of young adults born preterm: negative effects of rapid weight gain in early life. J Clin Endocrinol Metab (2012) 97:4498–506. 10.1210/jc.2012-1716 22993033

[11] Harder T Rodekamp E Schellong K Dudenhausen JW Plagemann A. Birth weight and subsequent risk of type 2 diabetes: a meta-analysis. Am J Epidemiol (2007) 165:849–57. 10.1093/aje/kwk071 17215379

[12] Gluckman PD Hanson MA. Living with the past: evolution, development, and patterns of disease. Science (2004) 305:1733–6. 10.1126/science.1095292 15375258

[13] Luo ZC Delvin E Fraser WD Audibert F Deal CI Julien P Maternal glucose tolerance in pregnancy affects fetal insulin sensitivity. Diabetes Care (2010) 33:2055–61. 10.2337/dc10-0819 20573751 PMC2928362

[14] Luo ZC Nuyt AM Delvin E Audibert F Girard I Shatenstein B Maternal and fetal IGF-I and IGF-II levels, fetal growth, and gestational diabetes. J Clin Endocrinol Metab (2012) 97:1720–8. 10.1210/jc.2011-3296 22419731

[15] Luo ZC Nuyt AM Delvin E Fraser WD Julien P Audibert F Maternal and fetal leptin, adiponectin levels and associations with fetal insulin sensitivity. Obesity (2013) 21:210–6. 10.1002/oby.20250 23505188

[16] Luo ZC Bilodeau JF Nuyt AM Fraser WD Julien P Audibert F Perinatal oxidative stress may affect fetal ghrelin levels in humans. Sci Rep (2015) 5:17881. 10.1038/srep17881 26643495 PMC4672324

[17] Schwarz NG Grobusch MP Decker ML Goesch J Poetschke M Oyakhirome S WHO 2006 child growth standards: implications for the prevalence of stunting and underweight-for-age in a birth cohort of Gabonese children in comparison to the Centers for Disease Control and Prevention 2000 growth charts and the National Center for Health Statistics 1978 growth references. Public Health Nutr (2008) 11:714–9. 10.1017/s1368980007001449 18167166

[18] Matthews DR Hosker JP Rudenski AS Naylor BA Treacher DF Turner RC. Homeostasis model assessment: insulin resistance and beta-cell function from fasting plasma glucose and insulin concentrations in man. Diabetologia (1985) 28:412–9. 10.1007/BF00280883 3899825

[19] Gonzalez Jimenez E [Body composition: assessment and clinical value]. Endocrinol Nutr (2013) 60:69–75. 10.1016/j.endonu.2012.04.003 22704270

[20] Austin PC Steyerberg EW. The number of subjects per variable required in linear regression analyses. J Clin Epidemiol (2015) 68:627–36. 10.1016/j.jclinepi.2014.12.014 25704724

[21] Soto N Bazaes RA Pena V Salazar T Avila A Iniguez G Insulin sensitivity and secretion are related to catch-up growth in small-for-gestational-age infants at age 1 year: results from a prospective cohort. J Clin Endocrinol Metab (2003) 88:3645–50. 10.1210/jc.2002-030031 12915649

[22] Kappeler L De Magalhaes Filho C Leneuve P Xu J Brunel N Chatziantoniou C Early postnatal nutrition determines somatotropic function in mice. Endocrinology (2009) 150:314–23. 10.1210/en.2008-0981 18801897

[23] Du Q Hosoda H Umekawa T Kinouchi T Ito N Miyazato M Postnatal weight gain induced by overfeeding pups and maternal high-fat diet during the lactation period modulates glucose metabolism and the production of pancreatic and gastrointestinal peptides. Peptides (2015) 70:23–31. 10.1016/j.peptides.2015.05.003 26022984

[24] Gungor N Saad R Janosky J Arslanian S. Validation of surrogate estimates of insulin sensitivity and insulin secretion in children and adolescents. J Pediatr (2004) 144:47–55. 10.1016/j.jpeds.2003.09.045 14722518

